# Supplementary material for: The gatekeeper of Yersinia type III secretion is under RNA thermometer control
Source: PLoS Pathog. 2021 Nov 12;17(11):e1009650. doi: 10.1371/journal.ppat.1009650 (PMC8612567; doi:10.1371/journal.ppat.1009650)
Supplement: S1 Table — (DOCX) [file ppat.1009650.s001.docx]

**Supporting information – S1 Table**

**S1 Table:** **Bacterial strains and plasmids used in this study.**

| **Strain or plasmid** | **Relevant genotype** | **Reference** |
| --- | --- | --- |
| ***Escherichia coli*** |  |  |
| S17-1 *λpir* | RP4-2 Tc::Mu-Km::Tn7 (*λpir*) | [1] |
| ***Yersinia pseudotuberculosis*** |  |  |
| YPIII | pIB1, wild type | [2] |
| YPIII Δ*yopN* | pIB1, Δ*yopN* | This study |
| **Plasmids** |  |  |
| pK18 | cloning vector, Kan^r^ | [3] |
| pBAD2-*bgaB*-His | reporter gene vector, *bgaB* with His-tag at the C-terminal end, P_BAD_ promotor, *araC,* Ap^r^ | [4] |
| pBAD2-*lcrF*-*bgaB*-His | reporter gene vector, translational fusion of *lcrF*(pYV0076)-RNAT and *bgaB* with His-tag at the C-terminal end, P_BAD_ promotor, *araC,* Ap^r^ | [4] |
| pBAD2-*gfp* | reporter gene vector, *gfp*, P_BAD_ promotor, *araC,* Ap^r^, Gm^r^ | [5] |
| pBAD2-*lcrF*-*gfp* | reporter gene vector, translational fusion of *lcrF*(PYV0076)-RNAT and *gfp*, P_BAD_ promotor, *araC,* Ap^r^, Gm^r^ | [6] |
| pDM4 | *sacBR, oriT*, *oriR6K*, Cm^r^ | [7] |
| pMK-*bla* | HindIII *sycE*-*yopE*_53_-BamHI-NLS-Cre-SalI fragment of pMK4 was replaced by a HindIII *sycE*-*yopE*_53_-BamHI-bla-SalI fragment of pBM53-Bla. Kan^r^ | [8] |
| pGM930 | pBAD24-Δ1 derivative, pHP45 tΩ terminator downstream of *araBp*, Ap^r^ | [9] |
| pBO6202 | pBAD2-*bgaB-*His, short 5’-UTR of *yopN* (pYP0065) plus 30 bp of the coding region, translational fusion | This study |
| pBO6203 | pBAD2-*bgaB-*His, long 5’-UTR of *yopN* (pYP0065) plus 30 bp of the coding region, translational fusion | This study |
| pBO6256 | pBAD2-*bgaB-*His, short 5’-UTR of *yopN* (pYP0065) plus 30 bp of the coding region, R1 variant (AAA13-15UCC), translational fusion | This study |
| pBO6257 | pBAD2-*bgaB-*His, short 5’-UTR of *yopN* (pYP0065) plus 30 bp of the coding region, R2 variant (A14C), translational fusion | This study |
| pBO6258 | pBAD2-*bgaB-*His, short 5’-UTR of *yopN* (pYP0065) plus 30 bp of the coding region, R3 variant (UG4-5CA), translational fusion | This study |
| pBO6216 | pBAD2-*bgaB-*His, short 5’-UTR of *yopN* (pYP0065) plus 30 bp of the coding region, D1 variant (C16A), translational fusion | This study |
| pBO6255 | pBAD2-*bgaB-*His, short 5’-UTR of *yopN* (pYP0065) plus 30 bp of the coding region, D2 variant (CG16,18AA) | This study |
| pBO6297 | pBAD2-*bgaB-*His, long 5’-UTR of *yopN* (pYP0065) plus 30 bp of the coding region, R1 variant (AAA78-80UCC), translational fusion | This study |
| pBO7802 | pBAD2-*bgaB-*His, 5’-UTR of *yscN* (pYP0067) plus 30 bp of the coding region, translational fusion | This study |
| pBO6029 | pBAD2-*bgaB-*His, short 5’-UTR of *yscA* (pYP00) plus 30 bp of the coding region, translational fusion | This study |
| pBO6022 | pBAD2-*bgaB-*His, long 5’-UTR of *yscA* (pYP00) plus 30 bp of the coding region, translational fusion | This study |
| pBO6207 | pBAD2-*gfp,* short 5’-UTR of *yopN* (pYP0065) plus 30 bp of the coding region, translational fusion | This study |
| pBO6269 | pBAD2-*gfp,* short 5’-UTR of *yopN* (pYP0065) plus 30 bp of the coding region, R1 variant (AAA13-15UCC), translational fusion | This study |
| pBO6268 | pBAD2-*gfp,* short 5’-UTR of *yopN* (pYP0065) plus 30 bp of the coding region, D2 variant (CG16,18AA), translational fusion | This study |
| pBO6247 | pK18, short 5’-UTR of *yopN* (pYP0065) plus 30 bp of the coding region, runoff plasmid for structure probing | This study |
| pBO6270 | pK18, short 5’-UTR of *yopN* (pYP0065) plus 30 bp of the coding region, runoff plasmid for structure probing, R1 variant (AAA13-15UCC) | This study |
| pBO6265 | pK18, short 5’-UTR of *yopN* (pYP0065) plus 60 bp of the coding region, runoff plasmid for primer extension inhibition | This study |
| pBO6273 | pK18, short 5’-UTR of *yopN* (pYP0065) plus 60 bp of the coding region, runoff plasmid for primer extension inhibition, R1 variant (AAA13-15UCC) | This study |
| pBO7408 | pDM4, *yopN* deletion fragment for generation of Δ*yopN* by bacterial conjugation | This study |
| pBO7423 | pGM930, short 5’-UTR and *yopN* (pYP0065) with a Strep-tag II at the C-terminal end | This study |
| pBO7440 | pGM930, short 5’-UTR and *yopN* (pYP0065) with a Strep-tag II at the C-terminal end, R1 variant (AAA13-15UCC) | This study |
| pBO7801 | pGM930, short 5’-UTR and *yopN* (pYP0065) with a Strep-tag II at the C-terminal end, D2 variant (CG16,18AA) | This study |

**References:**

1. Simon R, Priefer U, Pühler A. A broad host range mobilization system for *in vivo* genetic engineering: transposon mutagenesis in gram negative bacteria. Bio/Technology. 1983;1:784–791. doi:10.1038/nbt1183-784
2. Bölin I, Norlander L, Wolf-Watz H. Temperature-inducible outer membrane protein of *Yersinia pseudotuberculosis* and *Yersinia enterocolitica* is associated with the virulence plasmid. Infect Immun. 1982;37: 506–512. doi: 10.1128/IAI.37.2.506-512.1982
3. Pridmore RD. New and versatile cloning vectors with kanamycin-resistance marker. Gene. 1987;56: 309–312. doi:10.1016/0378-1119(87)90149-1
4. Righetti F, Nuss AM, Twittenhoff C, Beele S, Urban K, Will S, et al. Temperature-*responsive in vitro* RNA structurome of *Yersinia pseudotuberculosis*. Proc Natl Acad Sci. 2016;113: 7237–7242. doi:10.1073/pnas.1523004113
5. Roßmanith J, Narberhaus F. Exploring the modular nature of riboswitches and RNA thermometers. Nucleic Acids Res. 2016;44: 5410–5423. doi:10.1093/nar/gkw232
6. Twittenhoff C, Heroven AK, Mühlen S, Dersch P, Narberhaus F. An RNA thermometer dictates production of a secreted bacterial toxin. PLoS Pathog. 2020;16: e1008184. doi:10.1371/journal.ppat.1008184
7. Milton DL, O’Toole R, Horstedt P, Wolf-Watz H. Flagellin A is essential for the virulence of *Vibrio anguillarum*. J Bacteriol. 1996;178: 1310–1319. doi:10.1128/jb.178.5.1310-1319.1996
8. Köberle M, Klein-Günther A, Schütz M, Fritz M, Berchtold S, Tolosa E, et al. *Yersinia enterocolitica* targets cells of the innate and adaptive immune system by injection of Yops in a mouse infection model. PLoS Pathog. 2009;5: e1000551. doi:10.1371/journal.ppat.1000551
9. Delvillani F, Sciandrone B, Peano C, Petiti L, Berens C, Georgi C, et al. Tet-Trap, a genetic approach to the identification of bacterial RNA thermometers: application to *Pseudomonas aeruginosa*. RNA. 2014;20: 1963–1976. doi:10.1261/rna.044354.114
