# Supplementary material for: The gatekeeper of Yersinia type III secretion is under RNA thermometer control
Source: PLoS Pathog. 2021 Nov 12;17(11):e1009650. doi: 10.1371/journal.ppat.1009650 (PMC8612567; doi:10.1371/journal.ppat.1009650)
Supplement: S2 Table — (DOCX) [file ppat.1009650.s002.docx]

**Supporting information – S2 Table**

**S2 Table: Oligonucleotides used in this study.**

| **Primer** | **Sequence 5’ - 3’** | **Purpose** | **Plasmid** |
| --- | --- | --- | --- |
| yopN_short_fw | TTTGCTAGCAATTGTAATTATAAACTG | forward primer to amplify the short 5’-UTR of *yopN* (pYV0065) plus 30 bp of the coding region | pBO6202 pBO6207 |
| yopN_fw | TTTGCTAGCGCAGCACTAAAATTTCTC | forward primer to amplify the long 5’-UTR of *yopN* (pYV0065) plus 30 bp of the coding region | pBO6203 |
| yopN_rv | TTTGAATTCGCCATAAGATAGGTTATG | reverse primer to amplify the short or long 5’-UTR of *yopN* (pYV0065) plus 30 bp of the coding region | pBO6203 pBO6203 pBO6207 |
| yopN_R1_fw | AATTGTAATTATTCCCTGTTCATCTCAGGGAGTAGTTATG | forward primer for mutagenesis of *yopN* (pYV0065) 5’-UTR, R1 variant (AAA13-15UCC) | pBO6256 pBO6269 pBO6270 pBO6273 pBO6297 pBO7440 |
| yopN_R1_rv | CATAACTACTCCCTGAGATGAACAGGGAATAATTACAATT | reverse primer for mutagenesis of *yopN* (pYV0065) 5’-UTR, R1 variant (AAA13-15UCC) | pBO6256 pBO6269 pBO6270 pBO6273 pBO6297 pBO7440 |
| yopN_R2_fw | AATTGTAATTATACACTGTTCATCTCAGGGAGTAGTTATG | forward primer for mutagenesis of *yopN* (pYV0065) 5’-UTR, R2 variant (A14C) | pBO6257 |
| yopN_R2_rv | CATAACTACTCCCTGAGATGAACAGTGTATAATTACAATT | reverse primer for mutagenesis of *yopN* (pYV0065) 5’-UTR, R2 variant (A14C) | pBO6257 |
| yopN_R3_fw | AATCATAATTATAAACTGTTCATCTCAGGGAGTAGTTATG | forward primer for mutagenesis of *yopN* (pYV0065) 5’-UTR, R3 variant (UG4-5CA) | pBO6258 |
| yopN_R3_rv | CATAACTACTCCCTGAGATGAACAGTTTATAATTATGATT | reverse primer for mutagenesis of *yopN* (pYV0065) 5’-UTR, R3 variant (UG4-5CA) | pBO6258 |
| yopN_D1_fw | GATAAGCAAGAACTATTTTAATAATCGCGGTAATTGTAA | forward primer for mutagenesis of *yopN* (pYV0065) 5’-UTR, D1 variant (C16A) | pBO6216 |
| yopN_D1_rv | TTACAATTACCGCGATTATTAAAATAGTTCTTGCTTATC | reverse primer for mutagenesis of *yopN* (pYV0065) 5’-UTR, D1 variant (C16A) | pBO6216 |
| yopN_D2_fw | AATTGTAATTATAAAATATTCATCTCAGGGAGTAGTTATG | forward primer for mutagenesis of *yopN* (pYV0065) 5’-UTR, D2 variant (CG16,18AA) | pBO6255 pBO6268  pBO7801 |
| yopN_D2_rv | CATAACTACTCCCTGAGATGAATATTTTATAATTACAATT | reverse primer for mutagenesis of *yopN* (pYV0065) 5’-UTR, D2 variant (CG16,18AA) | pBO6255 pBO6268  pBO7801 |
| yopN_short_rnf_toe_fw | GAAATTAATACGACTCACTATAGGGAATTGTAATTATAAACTG | forward primer to amplify the 5’-UTR of *yopN* (pYV0065) with a T7 promotor for structure probing and primer extension inhibition | pBO6247 pBO6265 |
| yopN_short_rnf_rv | TTTGATATCGCCATAAGATAGGTTATG | reverse primer to amplify the 5’-UTR of *yopN* (pYV0065) for structure probing | pBO6247 |
| yopN_short_toe_rv | CTGCTGGTAGCGGCTCGCTGAC | reverse primer to amplify the 5’-UTR of *yopN* (pYV0065) for primer extension inhibition | pBO6265 |
| yopN_5’_fw | TTTGAGCTCAACCTGATGCATTGTCCCTG | forward primer to amplify the 5’-flank of *yopN* (pYV0065) for deletion plasmid | pBO7408 |
| yopN_5’_rv | AGATAGGTTATGAAGCGTCG | reverse primer to amplify the 5’-flank of *yopN* (pYV0065) for deletion plasmid | pBO7408 |
| yopN_3’_fw | CGACGCTTCATAACCTATCTGACCAAGTTAAAGGATTTTG | forward primer to amplify the 3’-flank of *yopN* (pYV0065) for deletion plasmid | pBO7408 |
| yopN_3’_rv | TTTGAGCTCTGTCGCACCATGTTGTTCCA | reverse primer to amplify the 3’-flank of *yopN* (pYV0065) for deletion plasmid | pBO7408 |
| yopN_external_fw | AACACCTGTCCCAGCAGATG | forward primer for confirmation of Δ*yopN* | - |
| yopN_external_rv | TAGCATCTTCACACCGGTGC | reverse primer for confirmation of Δ*yopN* | - |
| yopN_internal_fw | ATCGTAAATCAGACTCTGGG | forward primer for confirmation of Δ*yopN* | - |
| yopN_internal_rv | ATAACCCATCACTGCATCGC | reverse primer for confirmation of Δ*yopN* | - |
| yopN_strep_comp_fw | TTTCCATGGAATTGTAATTATAAACTGTTC | forward primer to amplify the short 5’-UTR of *yopN* (pYV0065) plus *yopN* with a Strep-II tag for complementation of Δ*yopN* | pBO7423 |
| yopN_strep_comp_rv | TTTCTGCAGTCATTTTTCGAACTGCGGGTGGCTCCAGAAAGGTCGTACGCCATTAG | reverse primer to amplify the short 5’-UTR of *yopN* (pYV0065) plus *yopN* with a Strep-II tag for complementation of Δ*yopN* | pBO7423 |
| yopN_short_qPCR_fw | CTGTTCATCTCAGGGAGTAG | forward primer to amplify the short *yopN* transcript for qRT-PCR analysis | - |
| yopN_short_qPCR_rv | ACGCTCGGAGAAGACAAATG | reverse primer to amplify the short *yopN* transcript for qRT-PCR analysis | - |
| yopN_long_qPCR_fw | TCGGCTGATTTTGGCATCGA | forward primer to amplify the long *yopN* transcript for qRT-PCR analysis | - |
| yopN_long_qPCR_rv | CTATCTGCACAGATTCTCCC | reverse primer to amplify the long *yopN* transcript for qRT-PCR analysis | - |
| gyrB_qPCR_fw | TCGCCGTGAAGGTAAAGTTC | forward primer to amplify the *gyrB* transcript for qRT-PCR analysis | - |
| gyrB_qPCR_rv | CGTAATGGAAGTGGTCTTCT | reverse primer to amplify the *gyrB* transcript for qRT-PCR analysis | - |
| nuoB_qPCR_fw | GATCCTCTCGAGCAACATG | forward primer to amplify the *nuoB* transcript for qRT-PCR analysis | - |

**S2 Table: Continued.**

| **Primer** | **Sequence 5’ - 3’** | **Purpose** | **Plasmid** |
| --- | --- | --- | --- |
| nuoB_qPCR_rv | TAAAGCAGGTTCCGGCCA | reverse primer to amplify the *nuoB* transcript for qRT-PCR analysis |  |
| yscN_fw | TTTGCTAGCGTTAATATTTCAACCATTA | forward primer to amplify the 5’-UTR of *yscN* (pYV0067) plus 30 bp of the coding region | pBO7802 |
| yscN_rv | TTTGAATTCATGATGAGGTATCTGATCTA | reverse primer to amplify the 5’-UTR of *yscN* (pYV0067) plus 30 bp of the coding region | pBO7802 |
| yscA_short_fw | TTGCTAGCTTAGATTGTGAAGATTCAATGGG | forward primer to amplify the short 5’-UTR of *yscA* (pYV0077) plus 30 bp of the coding region | pBO6029 |
| yscA_long_fw | TTGCTAGCATTGTTGATATTGCCATGGAAG | forward primer to amplify the long 5’-UTR of *yscA* (pYV0077) plus 30 bp of the coding region | pBO6022 |
| yscA_rv | TTGAATTCTGTTCTATGTTTCGTTGAAATTTG | reverse primer to amplify the 5’-UTR of *yscA* (pYV0077) plus 30 bp of the coding region | pBO6022  pBO6029 |
