# Supplementary material for: The gatekeeper of Yersinia type III secretion is under RNA thermometer control
Source: PLoS Pathog. 2021 Nov 12;17(11):e1009650. doi: 10.1371/journal.ppat.1009650 (PMC8612567; doi:10.1371/journal.ppat.1009650)
Supplement: S1 Fig — Sequence comparison of the yopN 5’-UTRs and 30 nucleotides of the coding region between Y. pseudotuberculosis, Y. pestis and Y. enterocolitica. Bold black nucleotides indicate the putative SD region and start codon while red nucleotides indicate sequence variations between Yersinia species. l: long transcript; s: short transcript. (DOCX) [file ppat.1009650.s003.docx]

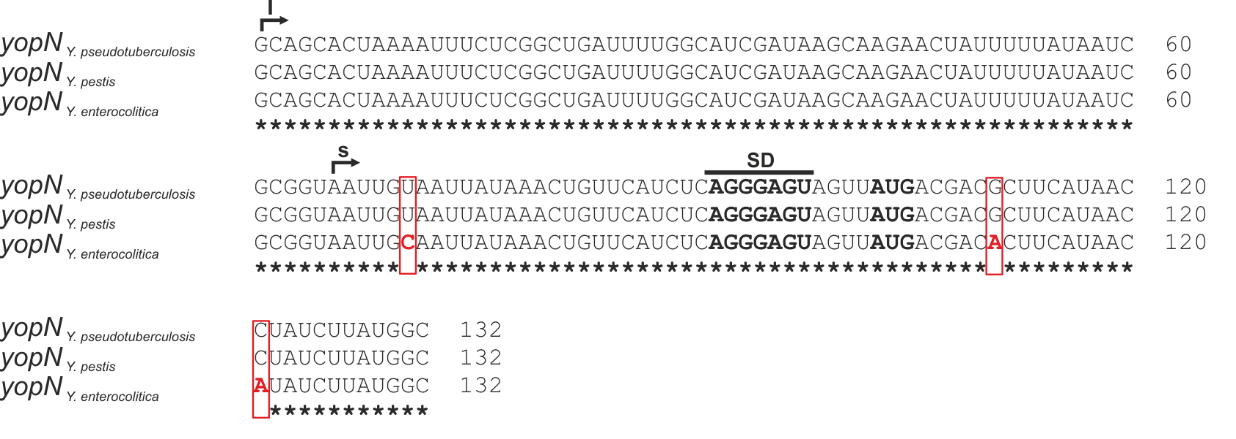


**S1 Fig. Sequence alignment of the *yopN* 5’-UTR of different *Yersinia* species.** Sequence comparison of the *yopN* 5’-UTRs and 30 nucleotides of the coding region between *Y. pseudotuberculosis*, *Y. pestis* and *Y. enterocolitica*. Bold black nucleotides indicate the putative SD region and start codon while red nucleotides indicate sequence variations between *Yersinia* species. l: long transcript; s: short transcript.
