# Supplementary material for: The gatekeeper of Yersinia type III secretion is under RNA thermometer control
Source: PLoS Pathog. 2021 Nov 12;17(11):e1009650. doi: 10.1371/journal.ppat.1009650 (PMC8612567; doi:10.1371/journal.ppat.1009650)
Supplement: S2 Fig — (A) Altered DNA fragment sizes due to yopN deletion using primers that bind within yopN (I:internal primers) and up- and downstream of the gene locus. Due to yopN deletion, no DNA fragment is produced with internal primers compared to the wild type (WT). L: DNA ladder; e: external primers; i: internal primers. (B) Schematic overview of primer localization. (DOCX) [file ppat.1009650.s004.docx]

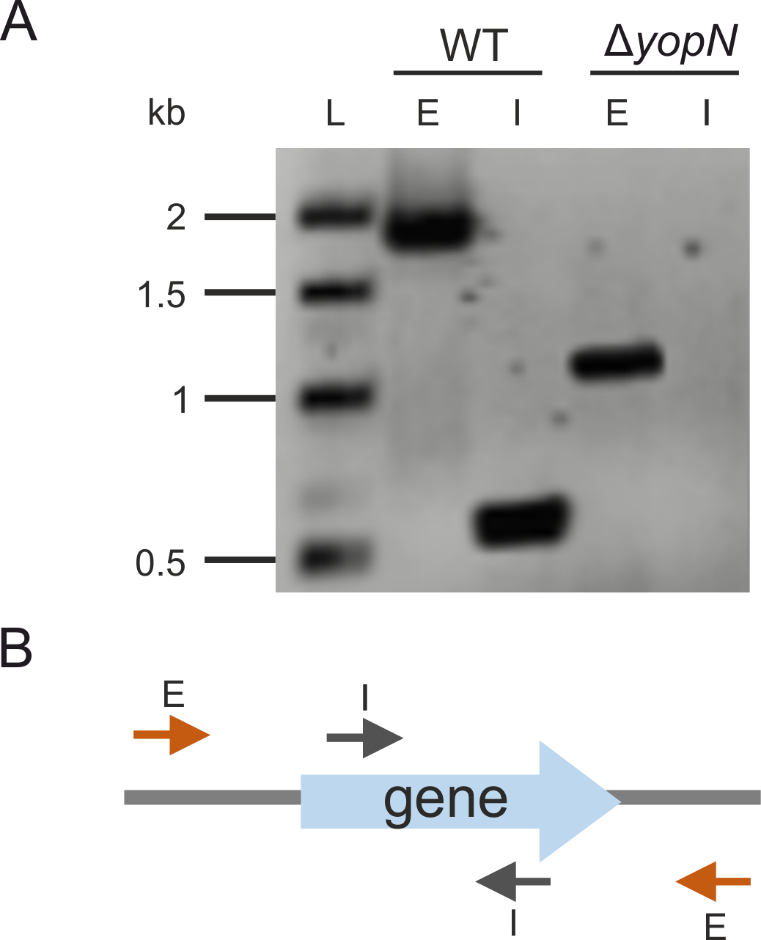


**S2 Fig. Confirmation of the Δ*yopN* mutant by PCR.** (A) Altered DNA fragment sizes due to *yopN* deletion using primers that bind within *yopN* (I:internal primers) and up- and downstream of the gene locus. Due to *yopN* deletion, no DNA fragment is produced with internal primers compared to the wild type (WT). L: DNA ladder; e: external primers; i: internal primers. (B) Schematic overview of primer localization.
