# Supplementary material for: The gatekeeper of Yersinia type III secretion is under RNA thermometer control
Source: PLoS Pathog. 2021 Nov 12;17(11):e1009650. doi: 10.1371/journal.ppat.1009650 (PMC8612567; doi:10.1371/journal.ppat.1009650)
Supplement: S3 Fig — (A) PARS-derived RNA structures of the 5’-UTRs of yscN (virB) and yscA (virC). The putative SD regions are highlighted in gray, the start codons in red. (B) Plasmid-based translational fusions of 5’-UTRs of interest and bgaB encoding a heat-stable β-galactosidase to test RNA thermometer (RNAT) functionality. Transcription of the fusion products is controlled by the arabinose-inducible promotor PBAD. The β-galactosidase assays of the short 5’-UTR of yopN (virA), the 5’-UTR of yscN (virB) and the short and long 5’-UTRs of yscA (virC) were conducted at 25 and 37°C. Y. pseudotuberculosis YPIII cells carrying plasmids of the fusion constructs were grown to an OD600 of 0.5 at 25°C. Subsequently, transcription of the reporter gene was induced by 0.1% (w/v) L-arabinose and the cultures were split to flasks at 25 and prewarmed flasks at 37°C and incubated for further 30 minutes. Samples were then taken for the β-galactosidase assay. The mean activities in Miller Units and the mean standard deviations were calculated from nine biological replicates. The representative Western blot displays the amount of BgaB-His produced. Protein amounts were adjusted to an optical density of 0.5 and detected by Ponceau S staining after blotting onto a nitrocellulose membrane. (DOCX) [file ppat.1009650.s005.docx]

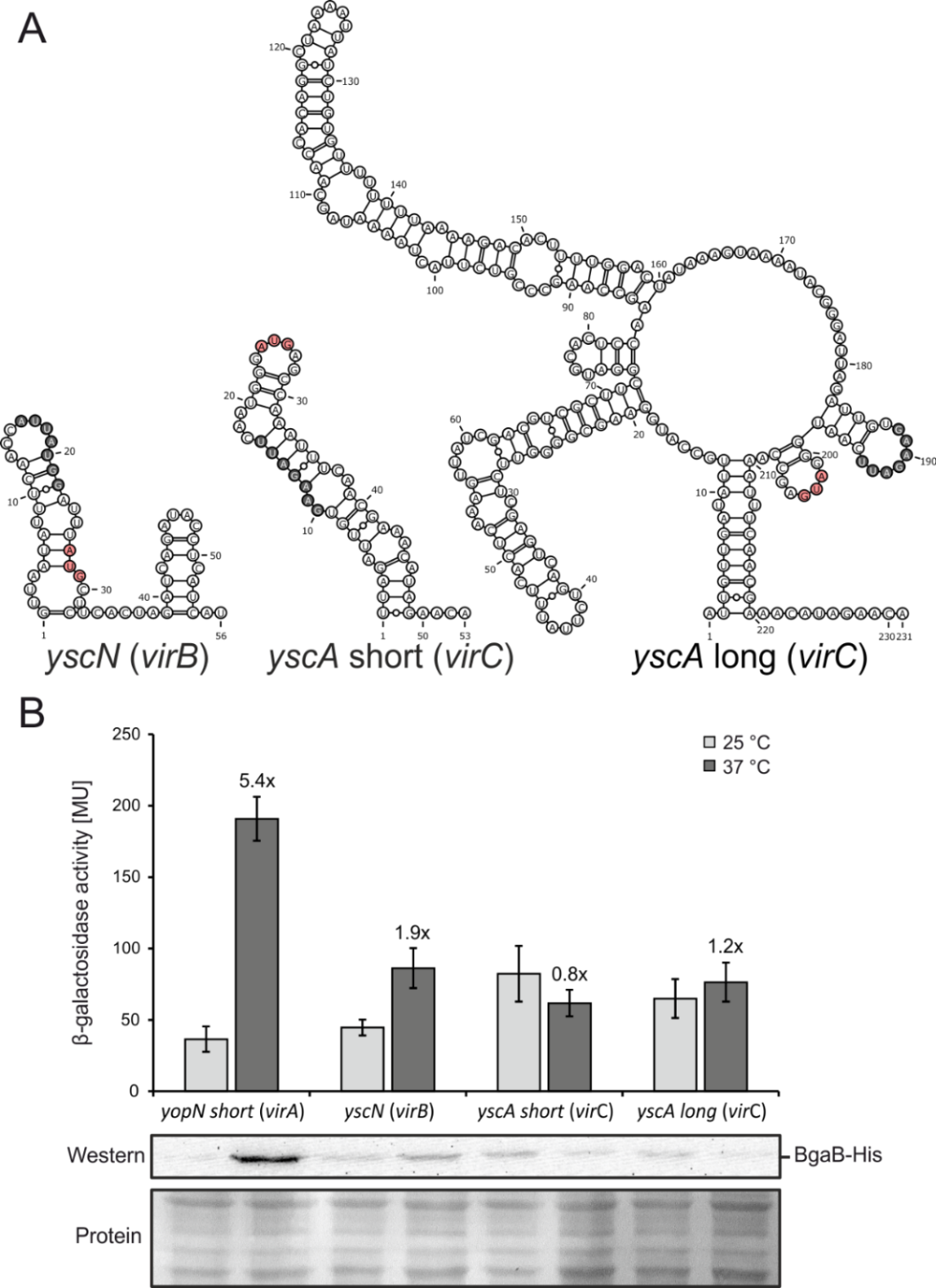


**S3 Fig.** **A functional RNAT is only present in the 5'-UTR of *virA* (*yopN*).** (A) PARS-derived RNA structures of the 5’-UTRs of *yscN* (*virB*) and *yscA* (*virC*) [1]. The putative SD regions are highlighted in gray, the start codons in red. (B) Plasmid-based translational fusions of 5’-UTRs of interest and *bgaB* encoding a heat-stable β-galactosidase to test RNA thermometer (RNAT) functionality. Transcription of the fusion products is controlled by the arabinose-inducible promotor P_BAD_. The β-galactosidase assays of the short 5’-UTR of *yopN* (*virA*), the 5’-UTR of *yscN* (*virB*) and the short and long 5’‑UTRs of *yscA* (*virC*) were conducted at 25 and 37 °C. *Y. pseudotuberculosis* YPIII cells carrying plasmids of the fusion constructs were grown to an OD_600_ of 0.5 at 25 °C. Subsequently, transcription of the reporter gene was induced by 0.1 % (w/v) L-arabinose and the cultures were split to flasks at 25 and prewarmed flasks at 37 °C and incubated for further 30 minutes. Samples were then taken for the β-galactosidase assay. The mean activities in Miller Units and the mean standard deviations were calculated from nine biological replicates. The representative Western blot displays the amount of BgaB-His produced. Protein amounts were adjusted to an optical density of 0.5 and detected by Ponceau S staining after blotting onto a nitrocellulose membrane.

**Reference:**

1. Righetti F, Nuss AM, Twittenhoff C, Beele S, Urban K, Will S, et al. Temperature-*responsive in vitro* RNA structurome of *Yersinia pseudotuberculosis*. Proc Natl Acad Sci. 2016;113: 7237–7242. doi:10.1073/pnas.1523004113
