# Supplementary material for: The gatekeeper of Yersinia type III secretion is under RNA thermometer control
Source: PLoS Pathog. 2021 Nov 12;17(11):e1009650. doi: 10.1371/journal.ppat.1009650 (PMC8612567; doi:10.1371/journal.ppat.1009650)
Supplement: S4 Fig — Comparison of relative transcript levels of endogenous expressed yopN in the wild type (WT) and induced yopN expression for strains carrying plasmid constructs (yopN, R1 and D2) under non-secretion (+ Ca2+) and secretion (- Ca2+) conditions at 25 and 37°C. Samples of Y. pseudotuberculosis YPIII strains were taken after 5 h followed by RNA isolation and qRT-PCR. Transcript levels were normalized to the amount of WT at 25°C under non-secretion conditions and to the reference genes nuoB and gyrB. The mean transcript amounts and standard deviations comprise the results of three biological replicates. Primer efficiencies were calculated by the CFX Maestro software (nuoB: 98.1%, gyrB: 95.4%, yopN short: 93.1%). (DOCX) [file ppat.1009650.s006.docx]

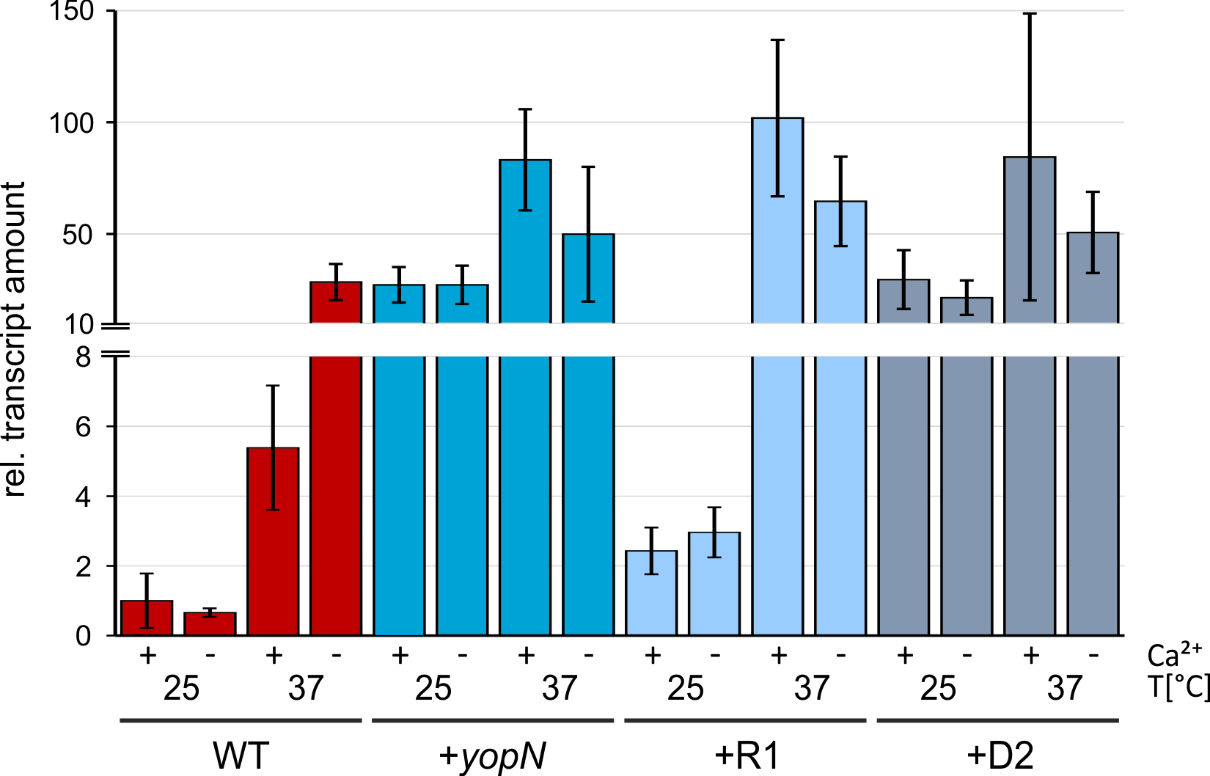


**S4 Fig. Transcript levels of *yopN* from cultures of the growth experiment shown in Fig 7.** Comparison of relative transcript levels of endogenous expressed *yopN* in the wild type (WT) and induced *yopN* expression for strains carrying plasmid constructs (*yopN*, R1 and D2) under non-secretion (+ Ca^2+^) and secretion (- Ca^2+^) conditions at 25 and 37 °C. Samples of *Y. pseudotuberculosis* YPIII strains were taken after 5 h followed by RNA isolation and qRT-PCR. Transcript levels were normalized to the amount of WT at 25 °C under non-secretion conditions and to the reference genes *nuoB* and *gyrB*. The mean transcript amounts and standard deviations comprise the results of three biological replicates. Primer efficiencies were calculated by the CFX Maestro software (*nuoB*: 98.1 %*, gyrB*: 95.4 %*, yopN* short: 93.1 %).
