# Supplementary material for: The gatekeeper of Yersinia type III secretion is under RNA thermometer control
Source: PLoS Pathog. 2021 Nov 12;17(11):e1009650. doi: 10.1371/journal.ppat.1009650 (PMC8612567; doi:10.1371/journal.ppat.1009650)
Supplement: S5 Fig — (A) The images show different cell morphologies of HEp-2 cells infected with Y. pseudotuberculosis YPIII wild type (WT) and different ΔyopN strains carrying either the empty vector (EV) or arabinose-inducible constructs of yopN with the wild type RNAT, the stable variant R1 or the open variant D2. Rounded HEp-2 cells indicate cytoskeleton-damaging activity of translocated YopE-TEM. Scale bars: 25 μm. All strains carry vector pMK-bla coding for a yopE-blaTEM fusion. HEp-2 cells were infected with bacterial strains at an MOI of 50. (B) Time course YopE translocation assay of HEp-2 cells infected with the aforementioned strains (MOI of 50) for 120 min at 37°C. Cells were labeled with CCF4-AM and the ratio between blue and green fluorescence (520 nm / 450 nm; normalized against uninfected cells) was measured every minute using the CLARIOstar Plus plate reader (BMG Labtech). Therefore, HEp-2 cells (2 x 10^4^) were seeded in 100 μL RPMI 1640 containing 7.5% NCS in 96-well plate and were cultivated in a 5% CO2 incubator at 37°C. Adherent cells were treated with CCF4-AM in the dark for 1 h at room temperature and the change of fluorescence was immediately measured after infection of Y. pseudotuberculosis strains at 37°C. (DOCX) [file ppat.1009650.s007.docx]

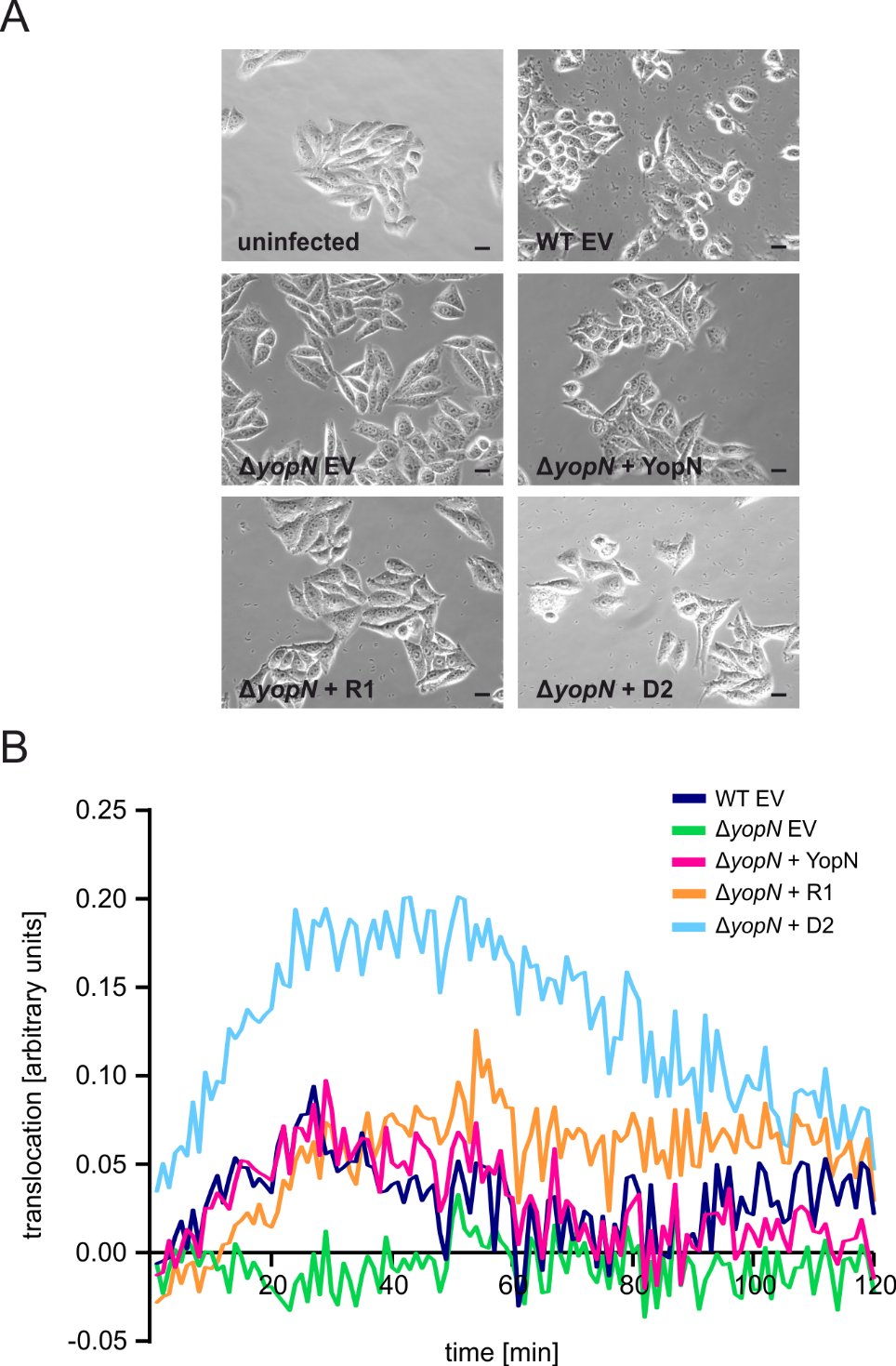


**S5 Fig. YopE translocation into HEp-2 cells.** (A) The images show different cell morphologies of HEp-2 cells infected with *Y. pseudotuberculosis* YPIII wild type (WT) and different Δ*yopN* strains carrying either the empty vector (EV) or arabinose-inducible constructs of *yopN* with the wild type RNAT, the stable variant R1 or the open variant D2. Rounded HEp-2 cells indicate cytoskeleton-damaging activity of translocated YopE-TEM. Scale bars: 25 µm. All strains carry vector pMK-*bla* coding for a *yopE*-*bla*_TEM_ fusion. HEp-2 cells were infected with bacterial strains at an MOI of 50.
(B) Time course YopE translocation assay of HEp-2 cells infected with the aforementioned strains (MOI of 50) for 120 min at 37 °C. Cells were labeled with CCF4-AM and the ratio between blue and green fluorescence (520 nm / 450 nm; normalized against uninfected cells) was measured every minute using the CLARIOstar Plus plate reader (BMG Labtech). Therefore, HEp‑2 cells (2 x 10^4^) were seeded in 100 µL RPMI 1640 containing 7.5 % NCS in 96-well plate and were cultivated in a 5 % CO_2_ incubator at 37 °C. Adherent cells were treated with CCF4-AM in the dark for 1 h at room temperature and the change of fluorescence was immediately measured after infection of *Y. pseudotuberculosis* strains at 37 °C.
